# Early T-cell reconstitution predicts risk of EBV reactivation after allogeneic hematopoietic stem cell transplantation

**DOI:** 10.1007/s10238-023-01270-3

**Published:** 2024-01-27

**Authors:** Jingtao Huang, Zengkai Pan, Luxiang Wang, Zilu Zhang, Jiayu Huang, Chuanhe Jiang, Gang Cai, Tong Yin

**Affiliations:** 1 Shanghai Institute of Hematology, State Key Laboratory of Medical Genomics, National Research Center for Translational Medicine at Shanghai, Ruijin Hospital, Shanghai JiaoTong University School of Medicine, Shanghai, China; 2 Department of Laboratory Medicine, Ruijin Hospital, Shanghai JiaoTong University School of Medicine, Shanghai, China

**Keywords:** Allo-HSCT, Myeloablative condition, Immune reconstitution, EBV reactivation, T cell

## Abstract

The quality of immune reconstitution (IR) is crucial for the outcome of patients who received allogeneic hematopoietic stem cell transplantation (allo-HSCT), and is closely connected with infection, relapse and graft-versus-host disease (GvHD) which are the most important causes for transplantation failure. However, the IR pattern in the early stage after allo-HSCT, particularly haploidentical (HID) HSCT, remains unclear. In this retrospective study, we examined the T cell reconstitution of patients within the initial 30 days (*n* = 173) and 100 days (*n* = 122) after allo-HSCT with myeloablative condition (MAC), of which > 70% were HID HSCT, to assess the influence of IR on the transplant outcomes. By comparing 78 patients with good IR (GIR) to 44 patients with poor IR (PIR), we observed that GIR was associated with lower risk for Epstein–Barr virus (EBV) reactivation and cytomegalovirus (CMV) reactivation, but had no significant impacts on the survival outcomes (i.e., overall survival, event-free survival) and cumulative incidences of GvHD. Importantly, we found lymphocyte reconstitution pattern at day 30 after allo-HSCT would be a surrogate for IR evaluated at day 100. In the Cox proportional hazard model, early reconstitution of CD4^+^, CD4^+^CD25^+^, CD4^+^CD45RO^+^, CD4^+^CD25^+^CD27^low^, and CD8^+^ T cells at day 30 was reversely correlated with risk of EBV reactivation. Finally, we constructed a predictive model for EBV reactivation with CD8^+^ and CD4^+^CD45RO^+^ T cell proportions of the training cohort (*n* = 102), which was validated with a validation cohort (*n* = 37). In summary, our study found that the quality of IR at day 30 had a predictive value for the risk of EBV reactivation, and might provide guidance for close monitoring for EBV reactivation.

**Supplementary Information:**

The online version contains supplementary material available at 10.1007/s10238-023-01270-3.

## Introduction

Allogeneic hematopoietic stem cell transplantation (allo-HSCT) is a potentially curative therapy for hematological malignancies. However, infection, relapse and graft-versus-host disease (GvHD) remain the major therapeutic challenges and affect the transplant outcomes. The quality of reconstitution of the donor-derived immune system in the recipient is of utmost importance for long-term survival after allo-HSCT [1–3]. Immune reconstitution (IR) would be completed in 2–5 years after allo-HSCT in a continuous and stepwise pattern, and this process would be affected by various factors [4]. Different kinds of immune cell subsets recover with different dynamics. After intensive conditioning, transplant recipients were in the “pre-engraftment phase” with prolonged neutropenia. Previous studies have confirmed that ATG_based prophylaxis are related to superior CD8^+^ T, γδ T, natural killer (NK) and NKT cell reconstitution, while the reconstitution of CD4^+^ T, regulatory T and B cell are faster in PTCy-based GvHD prophylaxis [5, 6] Neutrophil recovers within 14–30 days after graft infusion which defends against bacterial and fungal infections, which may be delayed after PTCy treatment, resulting more infections before day 100 [6]. The initial 100 days after transplantation are characterized by cellular immunodeficiencies due to a paucity of NK and T cells [7]. The compromised T cell reconstitution is primarily responsible for deleterious viral reactivations, including cytomegalovirus (CMV) and Epstein–Barr virus (EBV), as well as related viral end-organ diseases, rendering major reasons for morbidity and mortality for allo-HSCT [8].

Successful IR was defined as CD4^+^ T cells > 50 cells/μL in two consecutive measurements within 100 days after allo-HSCT [9, 10], which was associated with increased overall survival (OS) [9] and reduced non-relapse mortality (NRM) [10]. Besides, patients who received ATG treatment showed slower lymphocytes [11], CD4^+^ T, CD4^+^ CD25^+^ CD127^−^ T (Treg) and CD4^+^ CD25^−^ CD127^+^ T (Tconv) cell reconstitution [12] and higher rates of EBV reactivation [6], which indicated a potential association of EBV reactivation and CD4^+^ T subsets. However, the predictive value of CD4^+^ T cell recovery for the risk of viral reactivation is still weak [9, 10]. About 90% people in developing countries had primary EBV and CMV infections during childhood and adolescence [13]. When allo-HSCT was performed, the majority of EBV and CMV reactivations were usually observed within 3 months due to deficiency of normal cytotoxic T-cell monitoring [14, 15]. Persistent EBV reactivation is the most important risk factor for EBV-related post-transplant lymphoproliferative disorders (PTLD), resulting in dismal prognosis [16].

Furthermore, T-cell includes a panel of compartments, and CD4^+^ T-cell subset is not sufficient to recapitulate the status of cellular IR. Several lines of studies demonstrated the predictive value of other compartments of T-cells for transplant-related complications. Khandelwal et al. reported that CD38^bright^ CD8^+^ TEM > 35 cells/μL could predict acute GvHD (aGvHD) at a median of 8 days ahead of aGvHD onset [17]. Recent studies found that reduced numbers of total CD4^+^ T cells and naive CD4^+^ T cells at day 28 were significantly correlated with more infections [18]. Camargo et al. proposed that the absolute number of IL2^−^IFNγ^+^TNFα^−^MIP1β^+^CD8^+^T cells at a median of 30 days after allo-HSCT provided robust predictive value for risk of CMV reactivation [19]. Interestingly, Itzykson R et al. revealed that the CMV serostatus in recipient was positively correlated with the proportions of HLA-DR^+^ activated (CD8^+^HLA-DR^+^) and of late effector memory CD8^+^ (CD8^+^CD45RA^+^CCR7^−^) T cells [20]. Besides, successful EBV-specific immune responses are characterized by effective cytotoxic CD8^+^ T cells and NK cells [21, 22]. Higher proportion of CD8^+^ T cells had been observed in patients with EBV reactivation [15, 23]. After EBV reactivation, a sustained low proportion of CD4^+^ T cells was persistent within one year [24]. These findings suggested that numerous kinds of CD4^+^ and CD8^+^ cells participated in immune responses after allo-HSCT. However, detailed studies concerning the IR pattern at early stage after allo-HSCT, and its underlying significance for transplantation-related complications are still lacking.

In the present study, we retrospectively investigated the quality of IR at day 30 and day 100 after allo-HSCT, respectively, and constructed a predictive model with proportions of CD8^+^ and CD4^+^CD45RO^+^ populations at day 30 to predict EBV reactivation. Our data provided a systematic characterization of early T-cell reconstitution and analyzed its relationship with EBV reactivation.

## Patients and methods

### Study cohorts

This is a retrospective analysis based on the allo-HSCT database of Shanghai Ruijin Hospital. Consecutive adult patients receiving myeloablative allo-HSCT from April 2021 to April 2023 were screened, and the eligibility criteria were as follows: (1) age ≥ 16 years; (2) with a life expectancy ≥ 3 months; (3) receiving myeloablative condition; (4) during the first 3 months after allo-HSCT, the lymphocyte subsets were monitored at least once by flow cytometry. The last follow-up was July 30, 2023.

### Transplantation procedures

The protocol for the preconditioning regimen, GvHD prophylaxis and treatment, and infection prophylaxis were the same as previously reported [25, 26]. In brief, calcineurin inhibitors with short-term methotrexate and mycophenolate mofetil were served as the backbone for the GvHD prophylaxis. Among all patients included in this analysis, anti-thymocyte globulin (ATG), posttransplant cyclophosphamide (PTCy), a combination of ATG and PTCy [27], and a combination of Cyclosporin A (CSA) and Methotrexate (MTX) were adapted for 37, 15, 118 and 3 patients, respectively. The stem cell sources were granulocyte colony-stimulating factor (G-CSF) mobilized peripheral blood stem cell grafts from haploidentical related donors (*n* = 134), HLA-matched related donors (*n* = 22), and HLA-matched unrelated donors (*n* = 17).

### IR monitoring

After reaching neutrophil engraftment (> 500 cells/μL) following allo-HSCT, lymphocyte subsets were evaluated with flow cytometry at every week for up to 3 months. Cell acquisition was performed with a CantoII flow cytometer (BD Biosciences). All antibodies were titrated for optimum performance, and appropriate single-color compensation and fluorescence minus-one (FMO) controls were run. A time gate was initially drawn to ensure stable collection of samples. Cells in the “live” gate (Brilliant Violet 510-) were restricted by CD14- gate to remove monocytes and then by size (FSC) and granularity (SSC) to identify lymphocytes. Data were analyzed with FlowJo software, version 10.8.1.

### Monitoring for infections

CMV and EBV reactivation were monitored weekly by assessing plasma CMV and EBV DNA through real-time quantitative polymerase chain reaction (RT-qPCR) up to 1 year after allo-HSCT. We defined CMV and EBV DNA viremia as the detection of any level of CMV and EBV DNA in plasma samples. The EBV reactivation was defined as more than 1 × 10^3^ IU/mL EBV-DNA in plasma. CMV disease was diagnosed according to established criteria [28]. PTLD ﻿was defined by World Health Organization (WHO) classification of lymphoid neoplasms (2016 revision) [29]. The severity of infection was defined by Technical Manual of Procedures of Blood and Marrow Transplant Clinical Trials Network (version 3.0), consistent with previous study [30].

### Machine learning

Classification and regression tree (CART) machine-based learning was applied for EBV reactivation dichotomized as a binary outcome. The candidate factors for subgroup identification were summarized in Supplemental Table 1. All the variables have been established to correlate with allo-HSCT outcomes. For the EBV reactivation prediction model, lymphocyte subsets routinely monitored at day 30 after allo-HSCT were chosen as the median time of EBV activation was 60 (range: 25–355) days in our patients.

**Table 1:** Baseline characteristics of patients in immune reconstitution (IR) assessment cohort

	Total (*n* = 122)	GIR (*n* = 78)	PIR (*n* = 44)	*P* value
Median age, years (range)	43.5 (15–62)	43.5 (15–62)	44 (15–62)	0.8914
Gender, *n* (%)				0.2631
Male	58 (47.54)	34 (43.59)	24 (54.55)	
Female	64 (52.46)	44 (56.41)	20 (45.45)	
Follow-up duration in days, median (range)	381.5 (100–837)	385 (100–837)	340 (102–820)	0.6258
Underlying disease, *n* (%)				0.9739
AML	74 (23.77)	47 (60.26)	27 (61.36)	
ALL	29 (23.77)	18 (23.08)	11 (25.00)	
MDS	11 (9.02)	7 (8.97)	4 (9.09)	
Others	8 (6.56)	6 (7.69)	2 (4.55)	
HCT-CI scores before allo-HSCT, *n* (%)				0.7104
0 (low risk)	105 (86.06)	67 (85.90)	38 (86.36)	
1–2 (intermediate risk)	15 (12.30)	9 (11.54)	6 (13.64)	
≥ 3 (high risk)	2 (1.64)	2 (2.56)	0 (0)	
Donor type, *n* (%)				**0.0551**
HID	89 (72.95)	52 (66.67)	37 (84.09)	
Matched	33 (27.05)	26 (33.33)	7 (15.91)	
GvHD prophylaxis				**0.0051**
ATG	27 (22.13)	24 (30.77)	3 (6.82)	
PTCy	13 (10.66)	8 (10.26)	5 (11.36)	
ATG + PTCy	80 (65.57)	44 (56.41)	36 (81.82)	
CSA + MTX	2 (1.64)	2 (2.56)	2 (0)	
Blood group disparity, *n* (%)				0.1358
Matched	55 (45.08)	34 (43.59)	21 (47.73)	
Major mismatched	27 (22.13)	22 (28.21)	5 (11.36)	
Minor mismatched	25 (20.49)	13 (16.67)	12 (27.27)	
Major and minor mismatched	15 (12.30)	9 (11.53)	6 (13.64)	
MNC counts in graft, median (range, × 10^8^/kg)	12.11 (3.23–23.99)	11.78 (4.08–23.99)	12.49 (3.23–23.1)	0.9152
CD34^+^ cell counts in graft, median (range, × 10^6^/kg)	8.49 (2.17–15.6)	8.335 (2.98–15.6)	8.49 (2.17–14.9)	0.3713
Median time from HSCT to neutrophil engraftment (range)	13 (10–24)	13 (10–21)	13 (11–24)	0.8812
Median time from HSCT to platelet engraftment (range)	12 (9–27)	12 (9–25)	12 (10–27)	0.2402
Acute GvHD, *n* (%)	34 (27.87)	24 (30.77)	10 (22.73)	0.4039
Grade II-IV	20 (16.39)	13 (16.67)	7 (15.91)	> 0.9999
Grade III-IV	7 (5.74)	4 (5.13)	3 (6.82)	0.7019
Chronic GvHD, *n* (%)	32 (26.23)	21 (26.92)	11 (25.00)	> 0.9999
Infections after day 100, mean (range)	0.96 (0–13)	0.73 (0–6)	1.36 (0–13)	0.0595
Grade 3 infections, mean (range)	0.38 (0–7)	0.23 (0–2)	0.64 (0–7)	**0.0226**
CMV reactivation, *n* (%)	55 (45.08)	30 (38.46)	25 (56.82)	0.0596
Median time from HSCT to CMV reactivation (range)	41(10–110)	36 (10–75)	43 (21–110)	0.0871
CMV disease, n (%)	8 (6.56)	1 (1.28)	7 (15.91)	**0.0033**
EBV reactivation, *n* (%)	42 (34.43)	20 (25.64)	22 (50.00)	**0.0096**
Median time from HSCT to EBV reactivation (range)	60 (25–355)	58.5 (35–117)	63 (25–355)	0.5035
PTLD, n (%)	3 (2.46)	0 (0)	3 (6.82)	**0.0449**

GIR, Good immune reconstitution; PIR, Poor immune reconstitution; AML, acute myeloid leukemia; ALL, acute lymphocyte leukemia; MDS, myelodysplastic syndrome; HCT-CI, hematopoietic cell transplantation- specific comorbidity index; allo-HSCT, allogeneic hematopoietic stem cell transplantation; ATG, anti-thymocyte globulin; PTCy, posttransplant cyclophosphamide; CSA, Cyclosporin A; MTX, Methotrexate; HLA, human leukocyte antigen; MNC, mononuclear cells; GvHD, graft versus host disease; EBV, Epstein-Barr virus; PTLD, posttransplant lymphoproliferative disorders

### Statistical analysis

Chi-square or Fisher’s exact test was used in different categorical variables between two groups. Continuous variables were compared by unpaired two-tailed Student’s t test (for two group comparisons) or a one-way ANOVA. OS and event-free survival (EFS) were estimated using Kaplan–Meier curves and compared using the log-rank (Mantel-Cox) test. Cumulative incidence (CI) was used to determine the probability of relapse, CMV infection, EBV infection, aGvHD and chronic GvHD (cGvHD), and death as a competing risk. As the excellent protection of letermovir for CMV reactivation, only patients (*n* = 77) who did not receive letermovir as CMV prophylaxis were included in the analysis of the CIs of CMV reactivation. The frequency of infection was calculated as the average times of infection per patient.

Univariate logistics regression model was used to determine the predictive value of lymphocyte subsets at day 30 after allo-HSCT for the IR cohort. A *P-*value < 0.05 was considered statistically significant. To quantify the prediction performance of the lymphocyte subsets for IR quality, ﻿receiver operating characteristic (ROC) curve with calculated area under curve (AUC) was plotted. Univariate Cox regression model was used to determine prognostic factors for the training cohort (*n* = 102). Multivariate Cox regression analysis was performed on the variables that were statistically significant in the univariate analysis. The nomogram model for the risk of EBV reactivation was constructed by the final Cox regression analysis. The calibration curve was plotted to measure the calibration of the risk model. To quantify the prediction performance of the risk model, C-index was calculated via bootstrapping validation (1,000 bootstrap resamples), and the ROC curve was drawn to determine the best threshold for distinguishing the risk of EBV reactivation. The baseline characteristics of training and validation cohorts are summarized in Supplemental Table 2. All analyses were performed by statistical software R version 4.2.

**Table 2:** Different lymphocyte subsets of patients in GIR and PIR groups at day 30 after allo-HSCT

Subsets*	GIR median (range)	PIR median (range)	*P* value
CD3^+^ (%)	59.9 (0.4–93.2)	5.05 (0.1–98.6)	**< 0.0001**
CD4^+^ (%)	11.8 (0.4–50.6)	1.55 (0–35.1)	**< 0.0001**
CD8^+^ (%)	36.9 (0–85.7)	2.95 (0–92.9)	**0.0151**
CD4^+^/CD8^+^	0.42 (0–8.63)	0.36 (0–7.4)	0.6458
NK (%)	34.5 (4.1–98.8)	82.825 (1.4–98.8)	**0.0015**
CD19^+^ (%)	0.4 (0.1–7.01)	0.19 (0.04–25.8)	0.3870
CD4^+^CD25^+^ (%)	2 (0–14.8)	0.5 (0–11.2)	**0.0093**
CD4^+^CD45RA^+^ (%)	0.6 (0–13)	0.3 (0–31.6)	0.6174
CD4^+^CD45RO^+^ (%)	10.5 (0.1–49.2)	3.75 (0–35)	**0.0002**
CD4^+^CD25^+^CD127^low^ (%)	1.5 (0–13)	0.4 (0–9.2)	**0.0411**
CD3^+^HLA-DR^+^ (%)	27.4 (0.2–66.4)	7.95 (0–85.2)	0.0741
CD4^+^HLA-DR^+^ (%)	8.085 (0.09–22.66)	6.8 (0.26–20.39)	0.7825
CD4^+^CD25^+^CD127^−^HLA-DR^+^ (%)	0.5554 (0–4.712)	0.74855 (0.0271–8.4142)	0.2160
CD3^+^CD69^+^ (%)	2.5 (0.1–20)	1.25 (0–49.9)	0.7488
CD3^+^CD28^+^ (%)	41.53 (0.16–68.38)	1.27 (0–33.96)	**0.0398**
CD4^+^CD28^+^ (%)	12.05 (0.2–44.3)	4.4 (0–33)	**0.0002**
CD8^+^CD28^+^ (%)	14.4 (0.3–40.4)	6.85 (0.1–41.1)	0.4117
CD8^+^CD28^−^ (%)	25.65 (0.2–39.14)	8.465 (0.3–44.83)	0.5167
CD3^+^PD-1^+^ (%)	39.69 (0.42–67.14)	14.42 (0.83–30.21)	0.0860
CD4^+^PD-1^+^ (%)	10.3 (0.05–29.97)	2.605 (0.32–12.41)	**0.1539**
CD8^+^PD-1^+^ (%)	22.005 (0.21–64.92)	13.84 (0.34–37.24)	0.4649
CD4^+^CD45RA^+^CD27^+^ (%)	0.24 (0–4.75)	0.06 (0–0.2)	0.3436
CD4^+^CD45RA^+^CD27^−^ (%)	0.085 (0–0.84)	0.035 (0–0.48)	0.7381
CD4^+^CD45RA^−^CD27^+^ (%)	10.2 (0.11–23.7)	0.63 (0.16–12.97)	0.1110
CD4^+^CD45RA^−^CD27^−^ (%)	4.975 (0.11–19.52)	2.17 (0.04–4.5)	0.1830
CD8^+^CD45RA^+^CD27^+^ (%)	4.165 (0.18–22.47)	3.34 (0–20.13)	0.8015
CD8^+^CD45RA^+^CD27^−^ (%)	3.72 (0.38–12.45)	1.595 (0.22–25.6)	0.3922
CD8^+^CD45RA^−^CD27^+^ (%)	20.7 (0.11–37.82)	10.99 (0.26–30.5)	0.3723
CD8^+^CD45RA^−^CD27^−^ (%)	4.295 (0.07–24.17)	2.96 (0.04–11.6)	0.5535
CD8^+^CD45RO^+^ (%)	23.305 (0.11–58.29)	13.58 (0.26–38.71)	0.4457
CD19^+^IgD^+^CD27^−^ (%)	0.17 (0–3.28)	0.02 (0–36.74)	0.0946
CD19^+^CD20^−^CD38^+^ (%)	0.06 (0–0.71)	0.66 (0–6.8)	0.0620
CD19^+^IgD^+^CD27^+^ (%)	0.28 (0.01–0.46)	0.66 (0.17–1.79)	**0.0355**
CD19^+^IgD^−^CD27^+^ (%)	0.18 (0.03–0.63)	0.39 (0.12–0.66)	0.3016
CD19^+^CD27^+^CD38^+^ (%)	0.04 (0–0.41)	0.66 (0.06–36.32)	0.0769
CD20^+^ (%)	0.2 (0–3.5)	0.2 (0–43.5)	0.0773
TCR(ɑ/β) (%)	63.9 (0.3–90.1)	11.4 (0.1–90)	**0.0226**
TCR(γ/δ) (%)	2.5 (0–16.7)	0.8 (0–9.1)	0.1322
CD45RA^+^ (%)	27.1 (13.9–81.1)	50.9 (26.1–93.8)	**0.0311**
CD45RO^+^ (%)	65.5 (14.6–80.7)	40.2 (5.2–53.3)	**0.0040**
CD45RA^+^CD62L^+^ (%)	9.8 (2–43.7)	27.2 (13.6–63.7)	**0.0396**
CD45RO^+^CD62L^−^ (%)	25.5 (0.9–47)	12.9 (1.5–28)	**0.0405**

*The percentage of all subsets represents the proportion of total lymphocytes

## Results

### Patients’ characteristics and outcomes related to IR

**Fig. 1:**
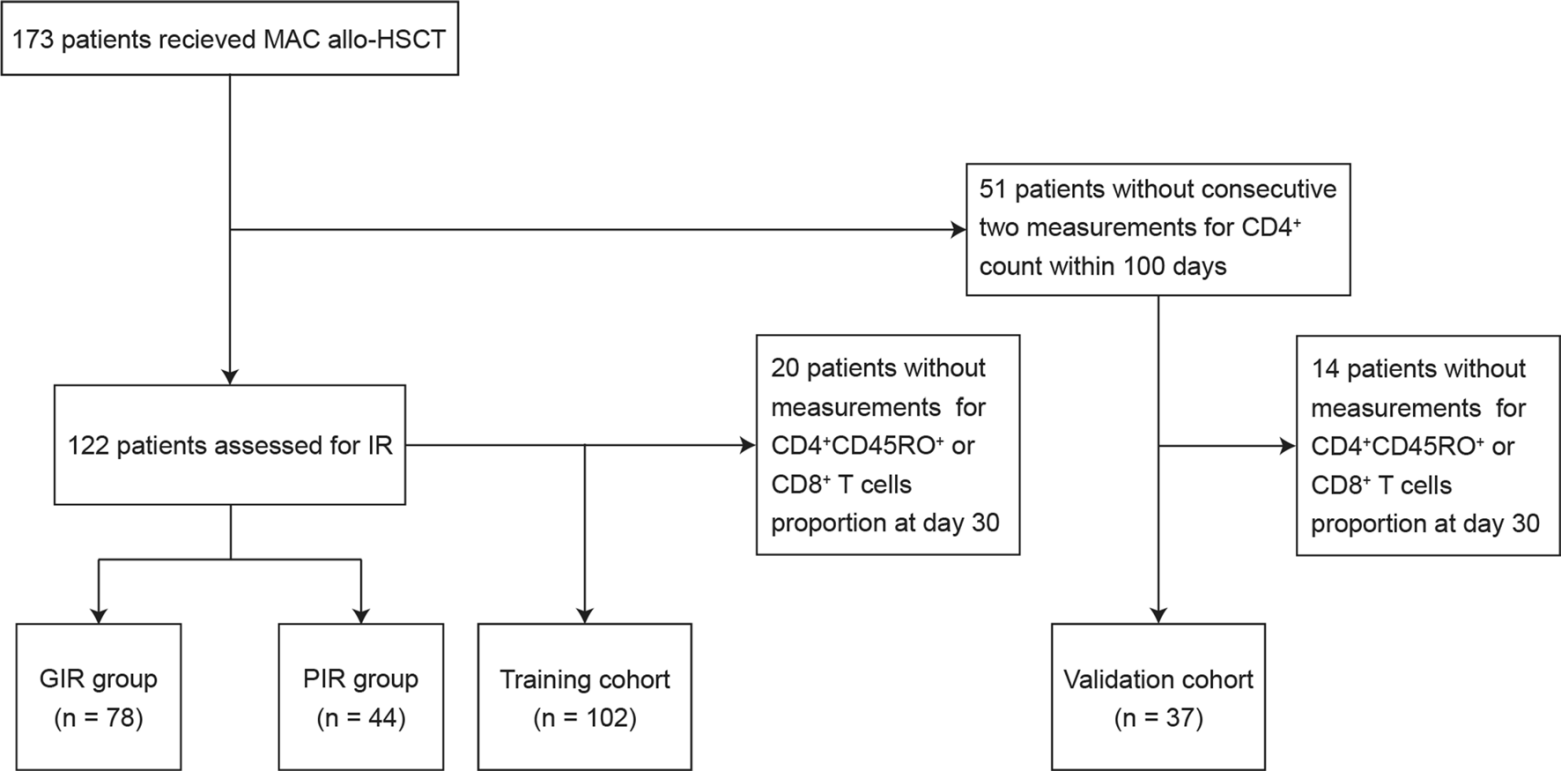
Patients included in this study

A total of 173 patients received MAC allo-HSCT with follow-up periods of more than 100 days were included in this study. One hundred and twenty-two patients were eligible for IR analysis, and 72.95% (89/122) of them received HID-HSCT. According to whether the patient achieved CD4^+^ T cells > 50 cells/μL in two consecutive measurements within 100 days after allo-HSCT [9], 78 patients were grouped as GIR and 44 patients were as PIR (Fig. 1). The median CD4^+^ T cell count for patients in GIR at day 30 was significantly higher than PIR (50 [1–465] vs. 4.5 [0–119] cells/μL, *P* < 0.0001) (Supplemental Fig. 1). Both groups were comparable in terms of most baseline transplant and disease characteristics, and the incidences of aGvHD and cGvHD. However, more patients in the GIR group received ATG as the GvHD prophylaxis (Table 1), which is consistent with previous studies [5, 6, 31].

Haploidentical transplantation was related to delayed CD3^+^ and CD4^+^ T cell reconstitution compared to matched transplantation since day 60 after allo-HSCT but did not affect the constitution of their subpopulations (Supplemental Fig. 2). Furthermore, we compared the IR of these two cohorts, and no statistical difference was observed (Supplemental Fig. 3A). The EBV reactivation rates of GIR and PIR patients in the haplo cohort and the matched cohort were comparable (Supplemental Fig. 3B). Except for the proportion of CD4^+^CD45RA^+^ T cells which recovered better in GIR patients of the matched cohort, no significant differences were found in the remaining subpopulations in either comparison group (Supplemental Fig. 3C and D).

**Fig. 2:**
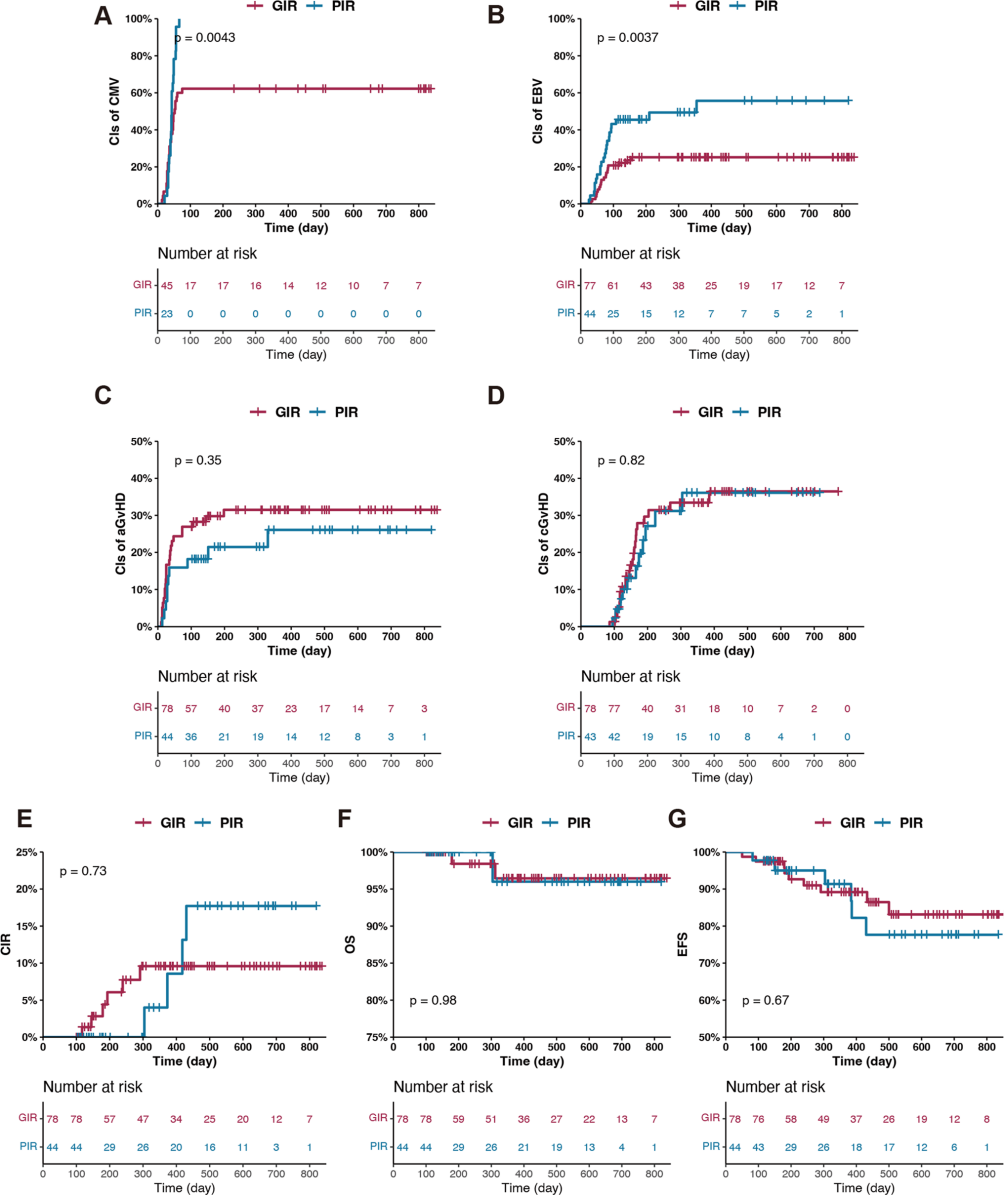
Outcome of patients in Good and Poor IR Group. CIs of CMV reactivation (**A**), EBV reactivation (**B**), aGvHD (**C**), cGvHD (**D**), and relapse (**E**) for different patients according to the quality of IR. Kaplan–Meier survival curve of OS (**F**) and EFS (**G**) for different patients according to quality of IR

**Fig. 3:**
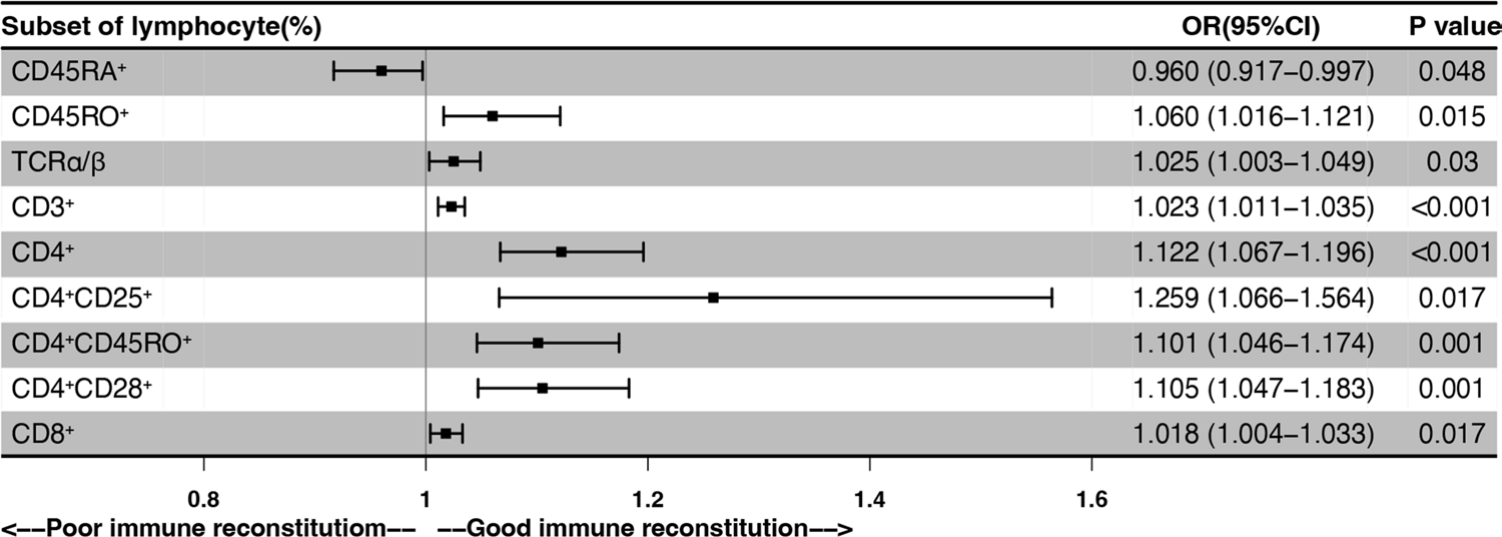
Logistics regression analysis. The outcome of IR up to day 100 after allo-HSCT, with the main lymphocyte subsets and absolute count at day 30

Compared to patients in GIR, patients in PIR suffered more frequently with grade 3 infections after day 100 (0.23 vs. 0.64, *P* = 0.0226) (Table 1). The CIs of CMV and EBV reactivation were much lower in patients from GIR group (62.2% vs. 100%, *P* = 0.0043; 25.2% vs. 55.7%, *P* = 0.0037) (Fig. 2A, B), as well as the incidences of CMV disease and PTLD (1.28% vs. 15.91% *P* = 0.0033; 0% vs. 6.82%, *P* = 0.0449) (Table 1). There were no statistically significant differences between the GIR and PIR groups in the CIs of acute/chronic GvHD and the survival outcomes (CIR, OS, EFS) (Fig. 2C–G).

### Early lymphocyte recovery at day 30 reflects IR quality

Since the median times for reactivation of CMV and EBV were 41 (range: 10–110) days and 60 (range: 25–355) days in our cohort after allo-HSCT, we wondered whether the lymphocyte recovery pattern at early stage after transplantation (i.e., day 30) could discriminate patients with GIR and PIR. The consecutive recovery of CD3^+^, CD4^+^, CD8^+^, CD4^+^CD25^+^ (Treg), CD4^+^CD25^+^CD127^low^ (Natural Treg, nTreg), and CD4^+^CD45RO^+^ T (CD4^+^ T memory) cells proportion were better in GIR group compared with their counterparts in PIR group (Supplemental Fig. 1). The differences had already appeared on day 30 after allo-HSCT (Table 2). The findings suggested that early lymphocyte recovery, especially CD4^+^ T-cell subpopulations at day 30 after allo-HSCT, may discriminate IR quality after allo-HSCT.

In univariate logistics analysis, we confirmed that higher proportions of CD3^+^, CD4^+^, CD4^+^CD25^+^, CD4^+^CD45RO^+^, CD4^+^CD28^+^, and CD8^+^ T subsets were associated with GIR (Fig. 3). Further, we examined the threshold of these subpopulations to distinguish IR status by ROC curve. Apart from the CD8^+^ T cell proportion, most of the other subsets had acceptable efficacy (AUC > 0.7). The most distinguishable T-cell compartments were the proportions of CD4^+^, CD4^+^CD28^+^, CD4^+^CD45RO^+^ and CD4^+^CD25^+^ (Supplemental Fig. 4), which were consistent with the result of univariant analysis.

**Fig. 4:**
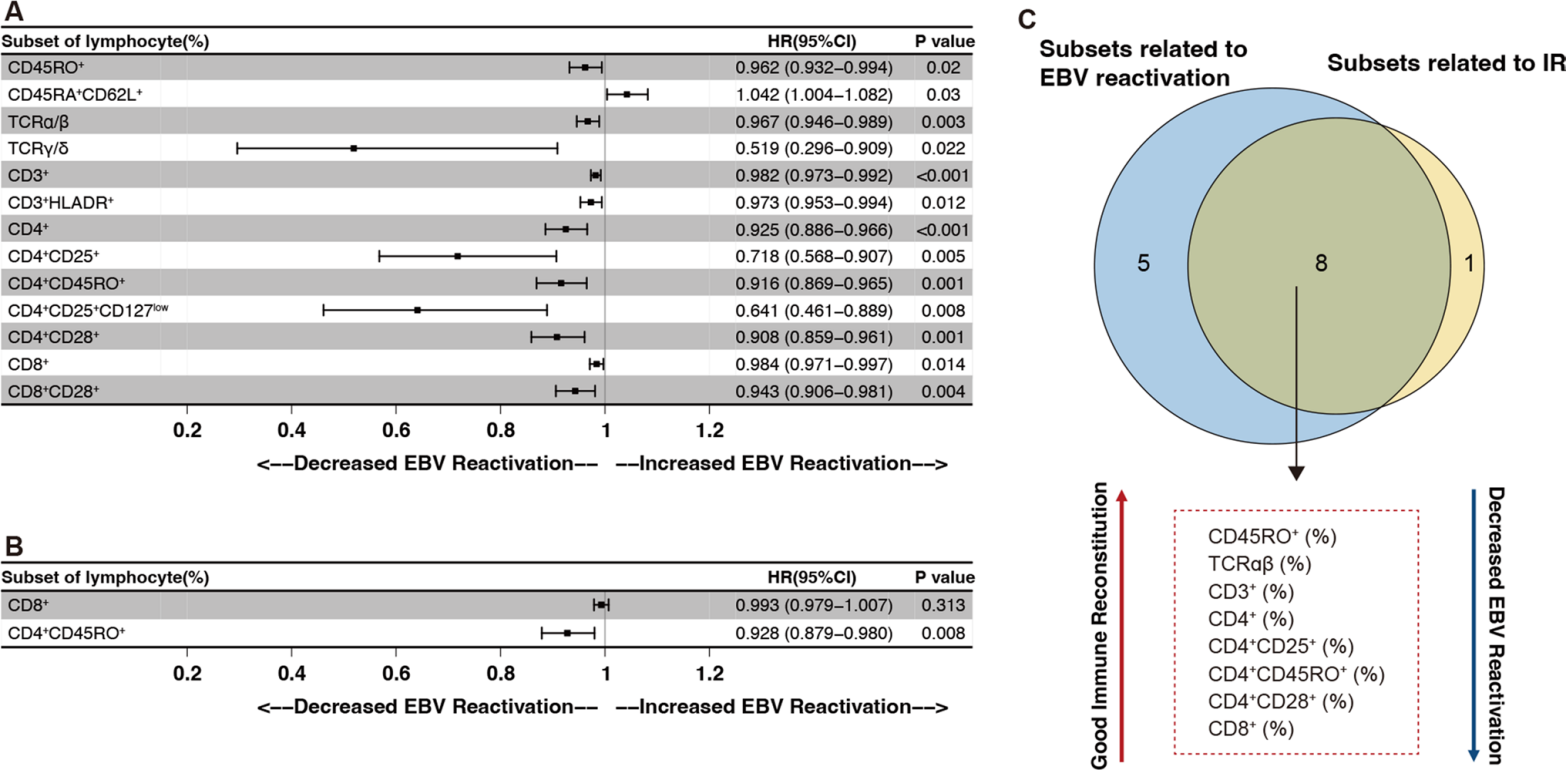
Association of EBV reactivation and with the early reconstitution of lymphocytes. Univariate analysis (**A**) and multivariate analysis (**B**) using a Cox proportional hazard model. The outcome of EBV reactivation after allo-HSCT, with the main lymphocyte subsets at day 30 day. (**C**) Venn diagram of lymphocyte subsets at day 30 after allo-HSCT affecting IR and EBV reactivation

### CD8^+^ and CD4^+^CD45RO^+^ reconstitution predicts EBV reactivation

Next, we tested whether the early T-cell reconstitution would predict the risk of EBV reactivation in all 173 patients. Compared to patients without EBV reactivation, the reconstitution of TCR(ɑ/β), TCR(γ/δ), CD3^+^, CD3^+^HLA-DR^+^, CD4^+^, CD4^+^CD25^+^, CD4^+^CD25^+^CD127^low^, CD4^+^HLA-DR^+^, CD4^+^CD45RO^+^, CD4^+^CD45RA^−^CD27^+^, CD8^+^ and CD8^+^CD28^+^ T cells at day 30 of patients with EBV reactivation was inferior (Supplemental Fig. 5). Univariate analysis confirmed that higher proportions of these subsets were associated with a decreased risk of EBV reactivation (Fig. 4A). Decreased proportions of CD4^+^CD25^+^, CD4^+^CD25^+^CD127^low^ and CD4^+^CD45RO^+^ T cells at day 30 were risk factors for EBV reactivation (Fig. 4A). In multivariate analysis, a higher CD4^+^CD45RO^+^ proportion at day 30 after allo-HSCT was associated with a lower risk of EBV reactivation (HR: 0.928, 95%CI [0.879–0.980], *P* = 0.008) (Fig. 4B).

**Fig. 5:**
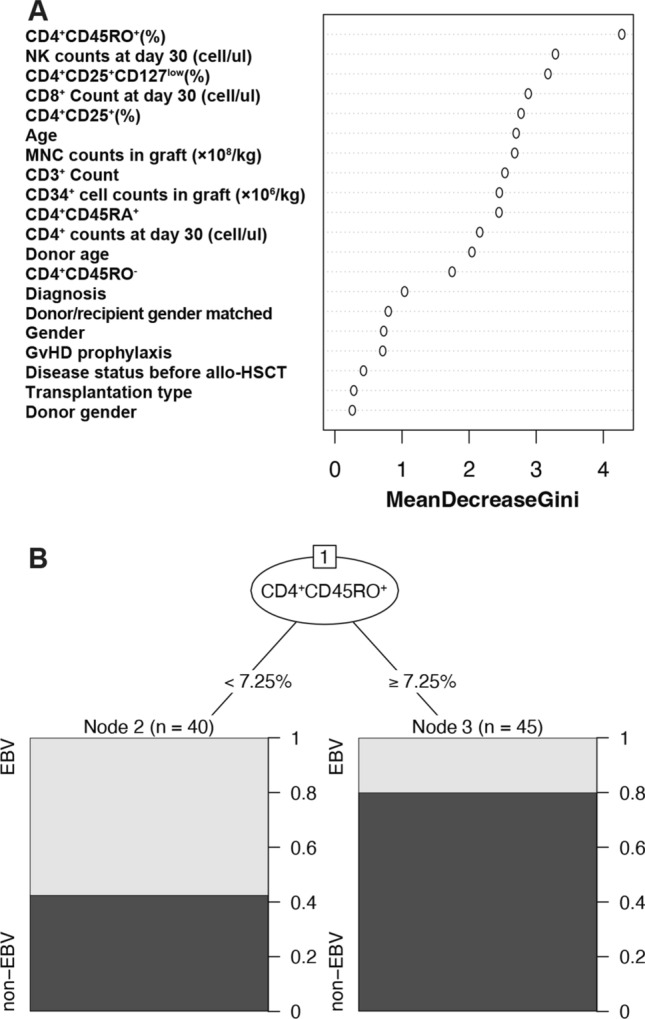
Classification for EBV reactivation by Machine learning. (**A**) The Gini index for various factors. (**B**) Flowchart of algorithm based on CART analysis for predicting EBV reactivation in our cohort

Venn diagram showed that lymphocyte subsets discriminating IR were shared with those in relation with EBV activation at day 30 after allo-HSCT. Higher proportions of CD45RO + , TCR(ɑ/β), CD3^+^, CD4^+^, CD4^+^CD25^+^, CD4^+^CD45RO^+^, CD4^+^CD28^+^, and CD8^+^ were positively associated with GIR and served as protective factors of EBV reactivation (Fig. 4C). These results showed that EBV reactivation was correlated with early T-cell IR after allo-HSCT.

### Construction of a predictive model for EBV reactivation

To address the importance of these subsets influencing EBV reactivation, we utilized CART analysis to calculate the weight of early lymphocyte subsets and clinical characteristics in association with EBV reactivation. Among all variables included in the machine learning, the proportion of CD4^+^CD45RO^+^ T cells at day 30 after allo-HSCT had the highest Gini index (Fig. 5A). When the percent of CD4^+^CD45RO^+^ cells was greater than 7.25%, the risk for EBV reactivation was significantly reduced (Fig. 5B). These results were consistent with the results of Cox regression analysis described above (Fig. 4A, B), suggesting that CD4^+^CD45RO^+^ T cells may be essential in controlling EBV reactivation.

**Fig. 6:**
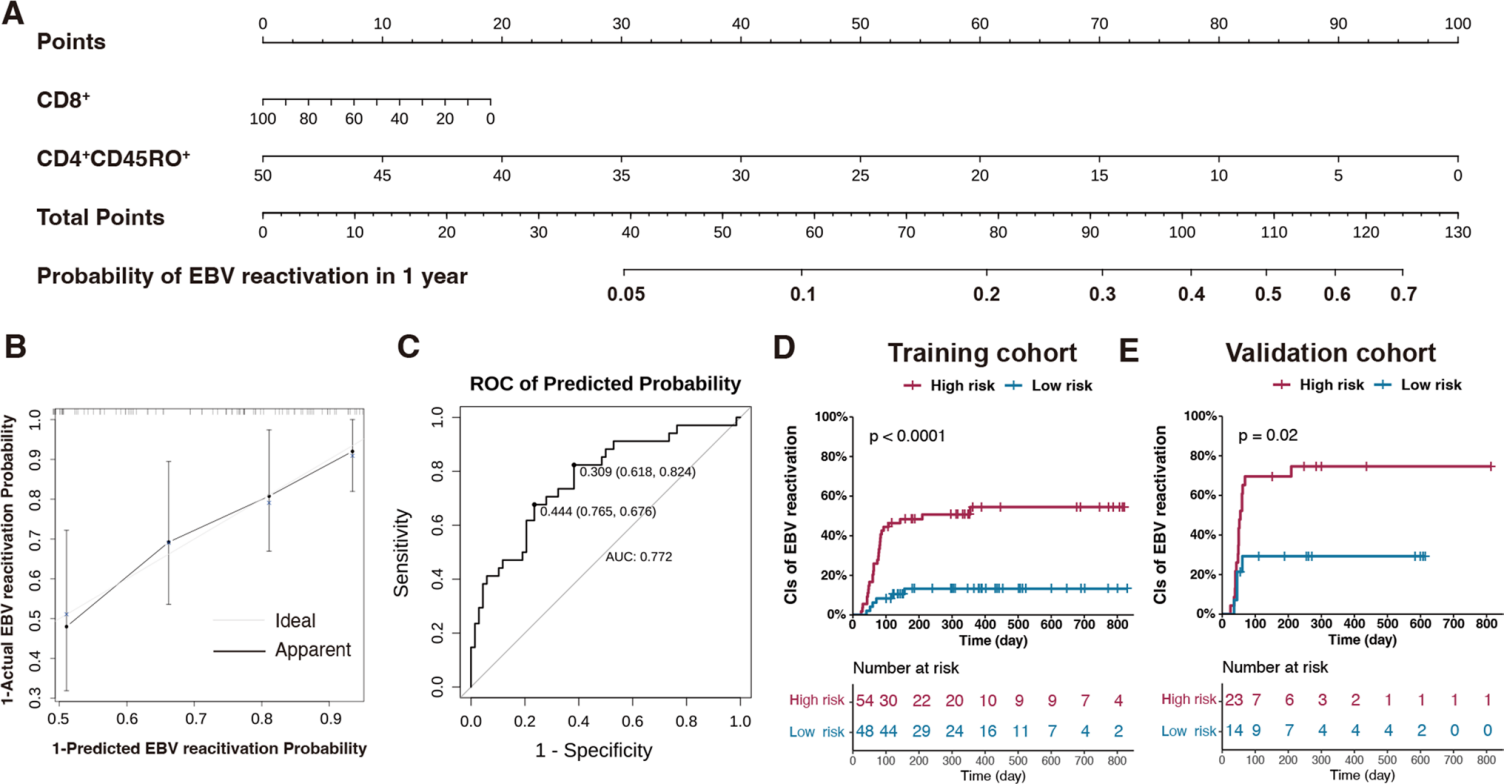
Early reconstitution of CD8^+^ and CD4^+^CD45RO^+^ T cells predict EBV reactivation within 1 year after allo-HSCT. (**A**) Nomogram model for EBV reactivation prediction of allo-HSCT patients with CD8^+^ and CD4^+^CD45RO^+^ T cells proportions at day 30. (**B**) Calibration plot of the predictive model. (**C**) ROC analysis of predicted probability for EBV reactivation. CIs of EBV reactivation of different patients in training cohort (IR cohort) (**D**) and validation cohort (**E**) based on the predicted probability of EBV reactivation. High risk: predicted probability > 0.309; Low risk: predicted probability ≤ 0.309

As multivariate analysis demonstrated that higher CD4^+^CD45RO^+^ proportion at day 30 after allo-HSCT was associated with lower risk of EBV reactivation and CD8^+^ T-cells were crucial in defense of EBV reactivation [32, 33], consistent with our univariant analysis (Fig. 4A), we developed a predictive model for EBV reactivation within 1 year after allo-HSCT by the Cox regression analysis with training cohort. Two key predictors, the proportions of CD8^+^ and CD4^+^CD45RO^+^ cells of total lymphocytes, were identified and presented in the nomogram (Fig. 6A). The calibration curve demonstrated that the nomogram had good concordance to predict the risk of EBV reactivation in this cohort (Fig. 6B). The C-index of the nomogram was 0.706, suggesting a good discriminative ability for this risk model. The AUC of ROC curve for this model was 0.772, which showed a high predictive value (Fig. 6C). Besides, using the predictive model, we divided patients in the training cohort into high-risk and low-risk groups (cut-off predictive probability > 0.309, which had higher specificity). The CIs of EBV reactivation of these two groups were 54.5% and 23.2% (*P* < 0.0001) (Fig. 6D), respectively.

To further verify the predictive value of the model, we screened 37 patients with measurements of CD8^+^ and CD4^+^CD45RO^+^ T cells at day 30 from 51 patients without complete IR data, as the validation cohort. According to the EBV reactivation predictive model, there were 23 and 14 patients each in the high-risk group and the low-risk group. The CIs of EBV reactivation of these two groups were 74.6% and 29.3% (*P* = 0.02) (Fig. 6E), respectively. These results indicated that early CD8^+^ and CD4^+^CD45RO^+^ T cell recovery at day 30 could predict the occurrence of EBV reactivation after allo-HSCT.

## Discussion

In this study, we retrospectively analyzed the association of immune cell recovery at day 30 and IR quality of 122 patients who received myeloablative allo-HSCT. As previous reported, CD4^+^ T cells reconstitution could predict OS in pediatric HSCT patients [9, 34], and better IR was associated with less viral reactivation [9]. In the present study, we confirmed that early recovery of T cell subsets at day 30 reflected IR quality in a cohort with more than 70% HID HSCT. And successful reconstitution of CD4^+^ T cells within 100 days was associated with lower CI of EBV reactivation. Several variables contribute to the increased risk of EBV reactivation after allo-HSCT. HLA mismatch is the most important risk factor for EBV reactivation after allo-HSCT [35–37]. Therefore, patients with HID HSCT would have a high risk for EBV reactivation. To note, we set the EBV-DNAemia (> 1000 IU/ml) as the diagnostic criterion for EBV reactivation according to the previous study [38]. As EBV-DNAemia accumulated, the risk of EBV-PTLD rose rapidly [38]. 27.40% (20/73) of patients with EBV reactivation in our cohort required rituximab intervention. Accurate prediction and close monitoring for EBV activation are warranted. Thus, we evaluated the T cell reconstitution at day 30 after allo-HSCT, and found that the lymphocyte reconstitution at day 30 effectively predicted EBV reactivation, which would be ahead of conventional IR evaluation timepoint (100 days post-transplantation).

Adaptive immunity is the core determinant for EBV prevention [39–41]. Several factors have a role in affecting immune recovery after allo-HSCT, spanning donor age, donor gender, intensive chemotherapy, GvHD prophylaxis, and conditioning regimens [42–45]. T cell recovery after allo-HSCT differs across individuals, and immune-monitoring might help to predict the risk of EBV reactivation shortly after allo-HSCT. ROC analysis revealed that the proportion of CD4^+^ and its subpopulation could distinguish GIR from PIR, while PIR patients are associated with higher incidence of EBV reactivation and PTLD, suggested that early CD4^+^ reconstitution may predict EBV infection post-transplantation. Our findings confirmed that impaired CD4^+^ T cell recovery was correlated with EBV reactivation and PTLD, which is consistent with previous studies [46, 47].

During primary EBV infection in healthy individuals, T cell numbers in peripheral blood were increased dramatically [39]. EBV-specific immune cells differentiated into memory CD4^+^ (including CD45RA^−^CD45RO^+^CCR7^−^ effector memory [EM] and CD45RA^−^CD45RO^+^CCR7^+^ central memory [CM] ~ 0.1%) and CD8^+^ (2–5%) T cells after infection [48]. For allo-HSCT recipients, early recovery of donor-derived EBV-specific T cells within 60 days provided prophylactic effects against EBV-related diseases [49]. The early recovery of the T cells relies on peripheral expansion of memory T cells, and CD8^+^ T cells reconstitute earlier than CD4^+^ T cells in early T-cell reconstitution. In the present study, CD8^+^ and CD4^+^CD45RO^+^ T cells within 30 days was reversely associated with EBV reactivation. Thus, our data suggested that CD8^+^ and CD4^+^CD45RO^+^ T cells after allo-HSCT provided a protection against EBV reactivation, possibly by driving early recovery of EBV-specific T cells. Furthermore, in the clinical setting, the criterion of the invention against EBV reactivation after allo-HSCT is not well established. Our data provided a promising method for risk-stratification of EBV reactivation, which might assist the judgment for early intervention.

Tregs is a subpopulation of CD4^+^ T cells with the function of suppressing immune responses and maintaining self-tolerance [50]. In our data, Tregs (CD4^+^CD25^+^ and CD4^+^CD25^+^CD127^low^ T cells) at day 30 after allo-HSCT were independent protective factors of EBV reactivation, which is consistent with previous studies that poor CD4^+^CD25^+^ T cell recovery at day 30 after allo-HSCT was associated with prolonged CMV and EBV duration [51, 52]. Taking the high inflammatory status of the early period after allo-HSCT into consideration, Tregs could have a compensatory increase and reflect high cytotoxic activity of effector T cells [53]. These findings suggested that a more careful evaluation of Tregs function in CMV/EBV reactivation is necessary, especially in the early period after allo-HSCT. Besides, there are many studies focus on the differential impact of CMV on outcome/immune reconstitution depending on CMV kinetics and higher CMV load is related to poor IR and clinical outcomes [5, 54, 55]. However, CMV reactivation after allo-HSCT is significantly limited in our cohort (data not shown) in the letermovir era, which is the reason that we're focused on predicting EBV activation with IR.

Our study has several limitations due to its retrospective design, small sample size, and short follow-up duration. Ongoing follow-up observation of survival in all patients is needed to verify the long-term effects of IR in HSCT patients. And the predictive model should be further evaluated by external cohorts. Thus, larger multicenter retrospective studies or prospective research endeavors are warranted.

In summary, our data suggested that early lymphocytes recovery, especially the CD4^+^ T cell and its subsets, were correlated with the quality of IR. We developed a prognostic nomogram for EBV reactivation based on the proportion of CD4^+^CD45RO^+^ and CD8^+^ T cells at day 30 after allo-HSCT, which may help to surveil the risk of EBV reactivation in early stages and intervene promptly.

## Supplementary Information

Below is the link to the electronic supplementary material.

Supplementary file1 (DOCX 25052 KB)
