# Supplementary material for: Early T-cell reconstitution predicts risk of EBV reactivation after allogeneic hematopoietic stem cell transplantation
Source: Clin Exp Med. 2024 Jan 27;24(1):22. doi: 10.1007/s10238-023-01270-3 (PMC10821970; doi:10.1007/s10238-023-01270-3)
Supplement: Supplementary file 1 — Supplementary file1 (DOCX 25052 KB) [file 10238_2023_1270_MOESM1_ESM.docx]

**Supplemental Appendix**

**Early T-cell reconstitution predicts risk of EBV reactivation after allogeneic hematopoietic stem cell transplantation**

Jingtao Huang^1 #^, Zengkai Pan^1 #^, Luxiang Wang^1^, Zilu Zhang^1^, Jiayu Huang^1^, Chuanhe Jiang^1^, Gang Cai^2*^, Tong Yin^1*^

**Affiliations:**

^1^ Shanghai Institute of Hematology, State Key Laboratory of Medical Genomics, National Research Center for Translational Medicine at Shanghai, Ruijin Hospital, Shanghai JiaoTong University School of Medicine, Shanghai, China

^2^ Department of Laboratory Medicine, Ruijin Hospital, Shanghai JiaoTong University School of Medicine, Shanghai, China.

^#^ These authors contributed equally.

*****Correspondence**:** yintong0101@163.com (T.Y); caigangsmmu@hotmail.com (G.C)

**Contents**

**Supplementary Figures**

**Supplemental Figure 1. The recovery trajectory of key lymphocyte subsets in GIR and PIR groups after allo-HSCT.**

**Supplemental Figure 2. The recovery trajectory of key lymphocyte subsets in haploidentical and matched transplantation groups.**

**Supplemental Figure 3. Comparison of the haploidentical and matched transplantation groups.**

**Supplemental Figure 4. ROC analysis of different key lymphocyte subsets at day 30 after allo-HSCT in GIR and PIR groups.**

**Supplemental Figure 5. Proportion of different T lymphocyte subsets at day 30 in patients with or without EBV reactivation after allo-HSCT.**

**Supplementary Tables**

Supplemental Table 1. Variables for building machine learning models.

Supplemental Table 2. Baseline characteristics of patients in training and validation cohort.

**Supplementary Figures**

**
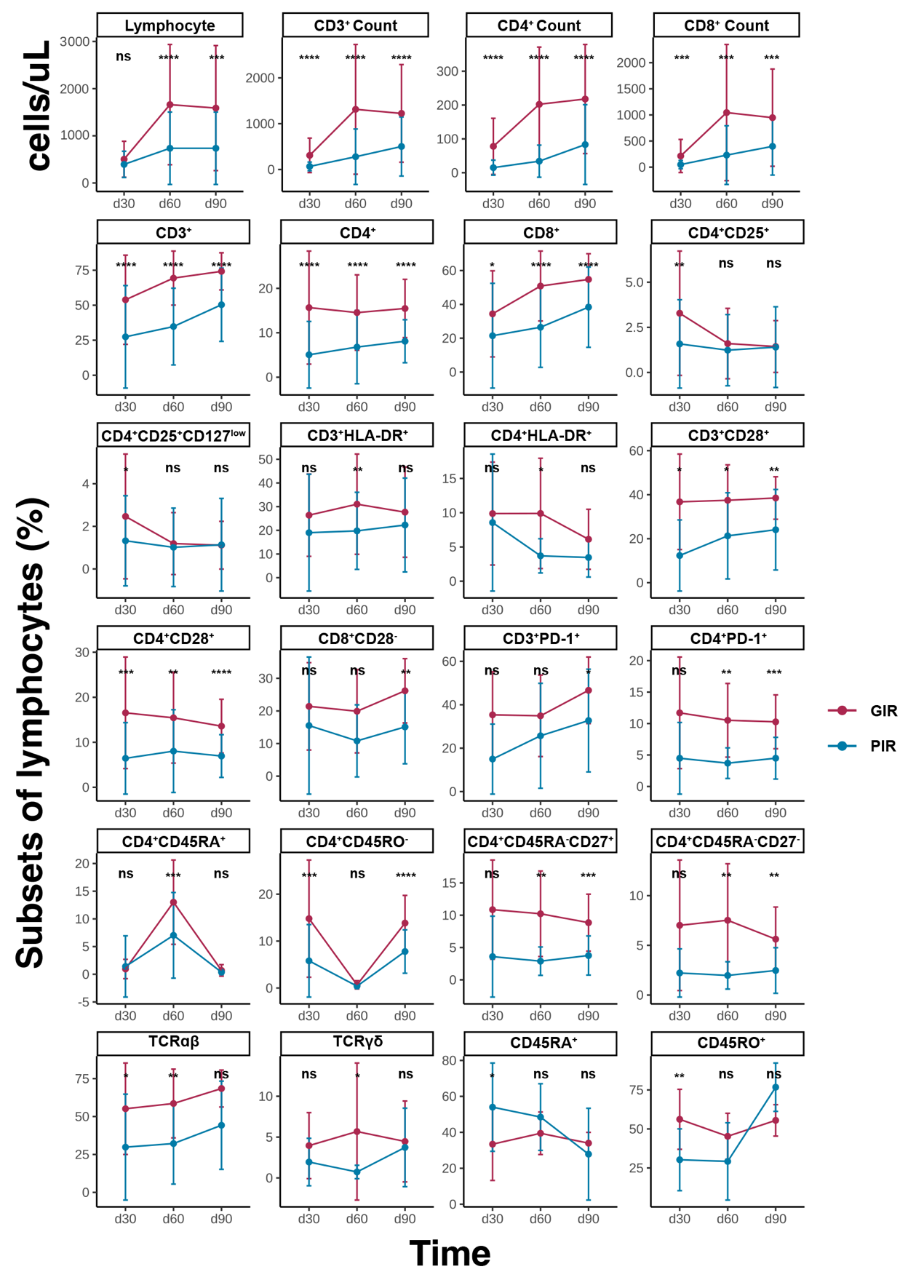
**

**Supplemental Figure 1. The recovery trajectory of key lymphocyte subsets in GIR and PIR groups after allo-HSCT.** Data was shown as mean ± SD. *p<0.05, **p<0.01, ***p<0.001, ****p<0.0001.

**
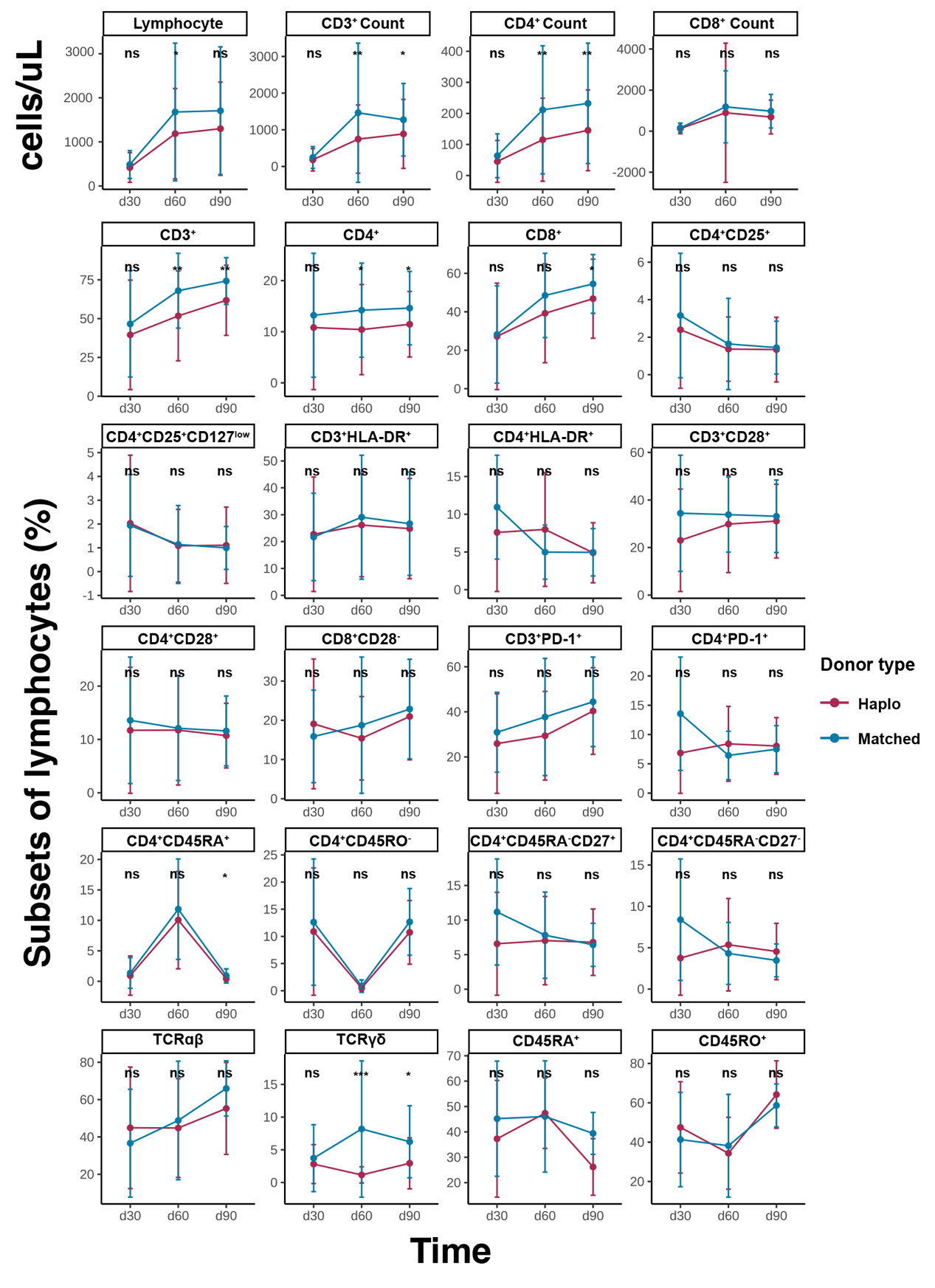
**

**Supplemental Figure 2. The recovery trajectory of key lymphocyte subsets in haploidentical and matched transplantation groups.** Data was shown as mean ± SD. *p<0.05, **p<0.01, ***p<0.001.

**
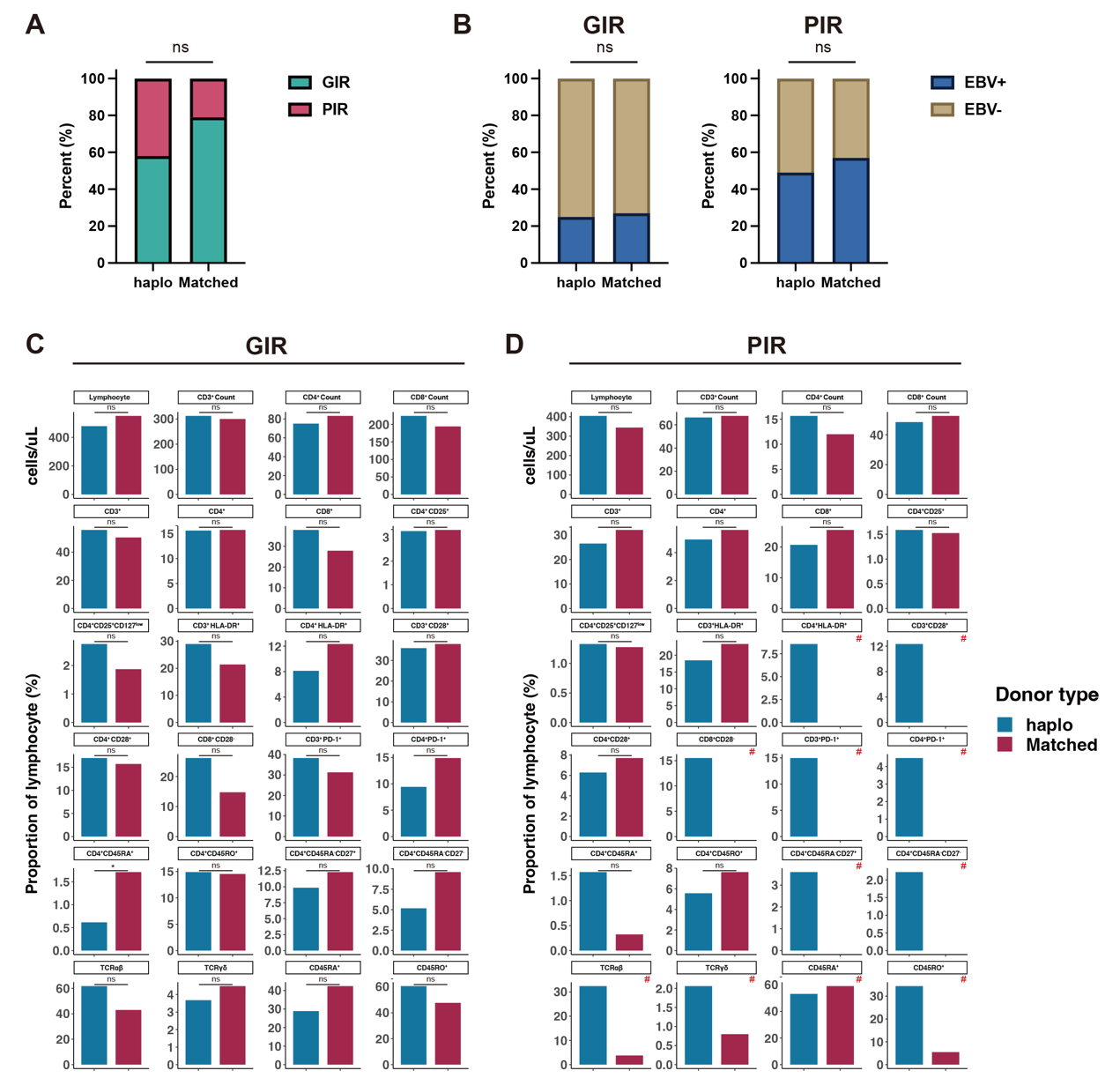
**

**Supplemental Figure 3. Comparison of the haploidentical and matched transplantation groups. (A)** The proportions of GIR and PIR patients in the haplo cohort and the matched cohort. **(B)** The proportion of EBV reactivation of GIR patients (left) and PIR patients (right) in the haplo cohort and the matched cohort. Comparison of early lymphocyte reconstitution of GIR patients **(C)** and PIR patients **(D)** in the haplo cohort and the matched cohort at day 30 after allo-HSCT. Data was shown as mean. *P < 0.05. #These subpopulations could not be analyzed for statistical differences due to lack of data.

**
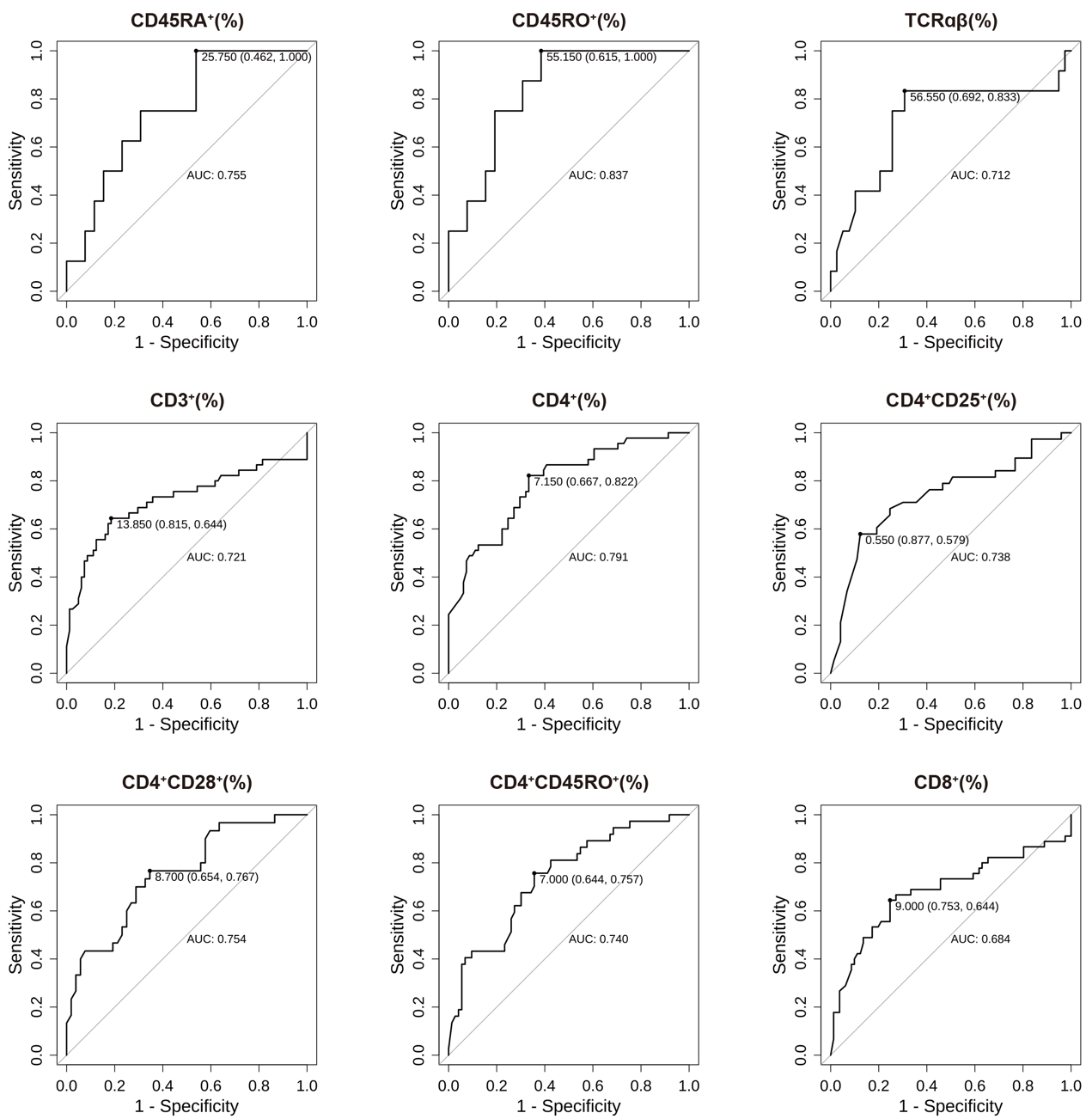
**

**Supplemental Figure 4. ROC analysis of different key lymphocyte subsets at day 30 after allo-HSCT in GIR and PIR groups.** AUC: Area under curve.

**
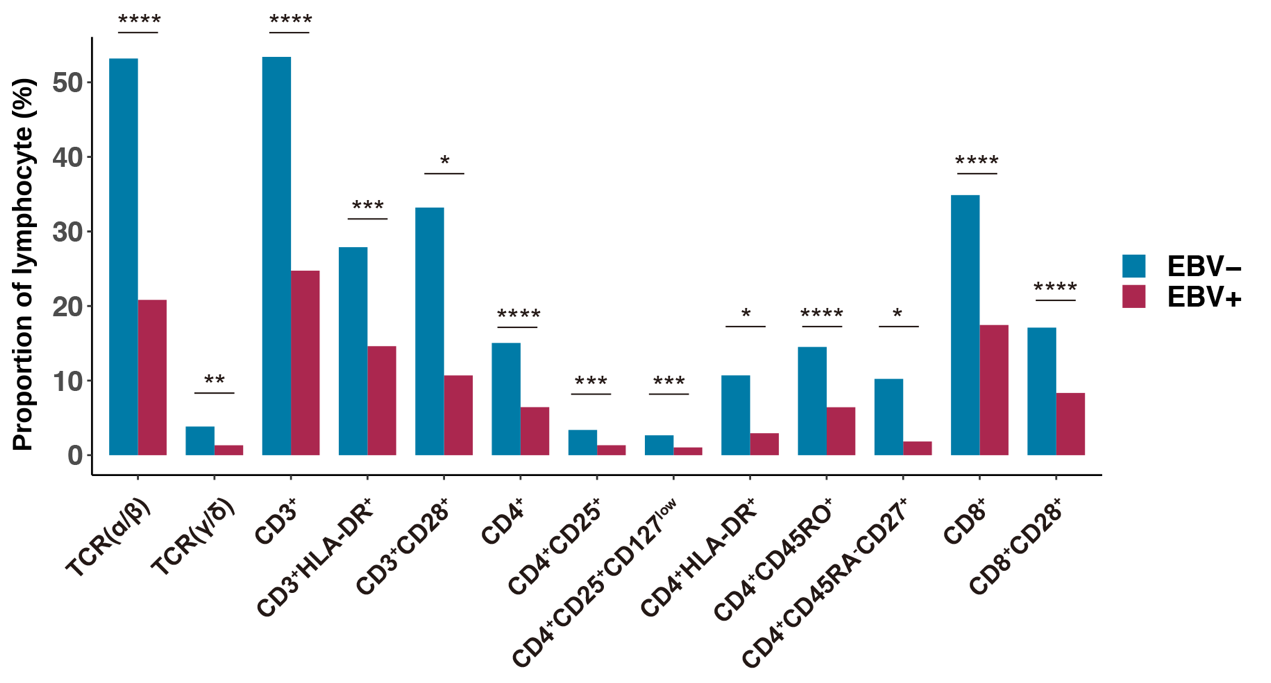
**

**Supplemental Figure 5. Proportion of different T lymphocyte subsets at day 30 in patients with or without EBV reactivation after allo-HSCT.** Data was shown as mean. *p<0.05, **p<0.01, ***p<0.001, ****p<0.0001.

**Supplementary Tables**

**Supplemental Table 1. Variables for building machine learning models**

| **Variables** | **Assignment for variables** |
| --- | --- |
| **Age (year)** | Numerical value |
| **Gender** | Male = 0; female = 1 |
| **Disease** | AML = 0; ALL = 1; MDS = 2; Others = 3 |
| **Disease status before allo-HSCT** | CR = 0; NR = 1; NA =2 |
| **GvHD prophylaxis** | ATG = 0; PTCy = 1; ATG+PTCy = 2; CSA+MTX =3 |
| **Donor type** | HID = 0; MSD = 1; MUD = 2; MMUD = 3; |
| **Donor age (year)** | Numerical value |
| **Donor gender** | Male = 0; female = 1 |
| **Donor/recipient gender matched** | Others = 0; female donor/male recipient = 1 |
| **MNC counts in graft (×10^8^/kg)** | Numerical value |
| **CD34^+^ cell counts in graft (×10^6^/kg)** | Numerical value |
| **CD3^+^ counts at day 30 (cell/ul)** | Numerical value |
| **CD4^+^ counts at day 30 (cell/ul)** | Numerical value |
| **CD8^+^ counts at day 30 (cell/ul)** | Numerical value |
| **NK counts at day 30 (cell/ul)** | Numerical value |
| **CD4^+^CD25^+^ (%)** | Numerical value |
| **CD4^+^CD45RA^+^ (%)** | Numerical value |
| **CD4^+^CD45RO^+^ (%)** | Numerical value |
| **CD4^+^CD45RO^-^ (%)** | Numerical value |
| **CD4^+^CD25^+^CD127^low^ (%)** | Numerical value |
| **EBV reactivation** | Negative = 0; positive = 1 |

Abbreviations: AML, acute myeloid leukemia; ALL, acute lymphocyte leukemia; MDS, myelodysplastic syndrome; allo-HSCT, allogeneic hematopoietic stem cell transplantation; CR, complete response; NR, non-response; ATG, anti-thymocyte globulin; PTCy, posttransplant cyclophosphamide; CSA, Cyclosporin A; MTX, Methotrexate; HID, haploidentical donor; MSD, matched sibling donor; MUD, matched unrelated donor; MMUD, mismatched unrelated donor; MNC, mononuclear cells; EBV, Epstein-Barr virus.

**Supplemental Table 2. Baseline characteristics of patients in training and validation cohort.**

|  | **Training cohort**  **(n=102)** | **Validation cohort**  **(n=37)** |
| --- | --- | --- |
| **Median age, years (range)** | 45 (15-62) | 51 (23-63) |
| **Gender, *n* (%)** |  |  |
| Male | 51 (50.00) | 18 (48.65) |
| Female | 51 (50.00) | 19 (51.35) |
| **Follow up duration in days,**  **median (range)** | 388.5 (100-830) | 309 (54-813) |
| **Underlying disease, *n* (%)** |  |  |
| AML | 62 (60.79) | 21 (56.76) |
| ALL | 27 (26.47) | 8 (21.62) |
| MDS | 8 (7.84) | 5 (13.51) |
| Others | 5 (4.90) | 2 (8.11) |
| **HCT-CI scores before allo-HSCT, *n* (%)** |  |  |
| 0 (low risk) | 87 (85.29) | 26 (70.27) |
| 1–2 (intermediate risk) | 13 (12.75) | 10 (27.03) |
| ≥ 3 (high risk) | 2 (1.96) | 1 (2.70) |
| **Donor type, *n* (%)** |  |  |
| HID | 77 (75.49) | 32 (86.49) |
| Matched | 25 (24.51) | 5 (13.51) |
| **GvHD prophylaxis** |  |  |
| ATG | 22 (21.57) | 7 (18.92) |
| PTCy | 13 (12.75) | 2 (5.41) |
| ATG+PTCy | 65 (63.73) | 28 (75.68) |
| CSA+MTX | 2 (1.96) | 0 (0) |
| **Blood group disparity, *n* (%)** |  |  |
| Matched | 50 (49.02) | 22 (59.46) |
| Major mismatched | 23 (22.55) | 10 (27.03) |
| Minor mismatched | 18 (17.65) | 3 (8.11) |
| Major and minor mismatched | 11 (10.78) | 2 (5.41) |
| **MNC counts in graft,**  **median (range, ×10^8^/kg)** | 12.28 (3.23-23.1) | 11.9 (6.33-25.63) |
| **CD34^+^ cell counts in graft,**  **median (range, ×10^6^/kg)** | 8.535 (2.17-15.6) | 9.27 (6.1-15.4) |
| **Median time from HSCT to neutrophil engraftment (range)** | 13 (10-24) | 13 (10-20) |
| **Median time from HSCT to platelet engraftment (range)** | 12 (9-27) | 13 (9-27) |
| **Acute GvHD, *n* (%)** | 31 (30.39) | 18 (48.65) |
| Grade Ⅱ-Ⅳ | 20 (19.61) | 13 (35.14) |
| Grade Ⅲ-Ⅳ | 7 (6.86) | 3 (9.09) |
| **Chronic GvHD, *n* (%)** | 25 (24.51) | 9 (24.32) |
| **Infections after day 100, mean (range)** | 1.09 (0-13) | 1.46 (0-8) |
| Grade 3 infections, mean (range) | 0.43 (0-7) | 0.41 (0-4) |
| **CMV reactivation, *n* (%)** | 49 (48.04) | 25 (67.57) |
| Median time from HSCT to CMV reactivation (range) | 42 (10-110) | 35 (11-135) |
| CMV disease, n (%) | 8 (7.84) | 3 (8.11) |
| **EBV reactivation, *n* (%)** | 34 (33.33) | 21 (56.76) |
| Median time from HSCT to EBV reactivation (range) | 67 (25-355) | 49 (25-209) |
| PTLD, n (%) | 2 (1.96) | 0 (0) |

Abbreviations: IR, Immune reconstitution; AML, acute myeloid leukemia; ALL, acute lymphocyte leukemia; MDS, myelodysplastic syndrome; HCT-CI, hematopoietic cell transplantation- specific comorbidity index; allo-HSCT, allogeneic hematopoietic stem cell transplantation; HID, haploidentical donor; ATG, anti-thymocyte globulin; PTCy, posttransplant cyclophosphamide; CSA, Cyclosporin A; MTX, Methotrexate; HLA, human leukocyte antigen; MNC, mononuclear cells; GvHD, graft versus host disease; EBV, Epstein-Barr virus; PTLD, posttransplant lymphoproliferative disorders;
